# Artificial intelligence-based personalized treatment strategies for unresectable hepatocellular carcinoma: integrating HSP90α for prognosis and survival prediction

**DOI:** 10.1038/s41746-025-02281-y

**Published:** 2025-12-27

**Authors:** Ke Su, Xin Liu, Xuelian Wang, Yunwei Han, Huiyan Luo, Lianbin Wen, Jian Chen, Han Li, Susu Xiao, Jianwen Zhang, Chenjie Wang, Yuhang Zhou, Zunyuan Tan, Lexin Wang, Peng Wang, Haiqing Chen, Guixu Zhang, Kun He, Xiaosong Li, Hao Chi, Zhenjiang Li, Ke Xu

**Affiliations:** 1 Western Institute of Digital-Intelligent Medicine, Chongqing, 401329 China; 2 Department of Oncology, The Affiliated Traditional Chinese Medicine Hospital, Southwest Medical University, Luzhou, 646000 China; 3 Department of Radiation Oncology, National Cancer Center/National Clinical Research Center for Cancer/Cancer Hospital, Chinese Academy of Medical Sciences and Peking Union Medical College, Beijing, 100021 China; 4 Department of Radiation Physics, Shandong Cancer Hospital and Institute, Shandong First Medical University and Shandong Academy of Medical Sciences, Jinan, 250117 China; 5 Department of Gynecological Radiotherapy, Harbin Medical University Cancer Hospital, Harbin, 150081 China; 6 Department of Oncology and Hematology, Zhongxian People’s Hospital, Chongqing, 404300 China; 7 Department of Oncology, The Affiliated Hospital of Southwest Medical University, Luzhou, China; 8 Department of Oncology, Chongqing General Hospital, Chongqing University, Chongqing, 401147 China; 9 Department of Geriatric Cardiology, Sichuan Academy of Medical Sciences & Sichuan Provincial People’s Hospital, Chengdu, 610072 China; 10 Department of Laboratory Medicine, The Second Affiliated Hospital of Chongqing Medical University, Chongqing, People’s Republic of China; 11 West China Hospital, Sichuan University, Chengdu, 610041 China; 12 Department of Oncology, Southwest Hospital, Third Military Medical-University (Army-Medical-University), Chongqing, 400038 China; 13 Department of Clinical Medical College, Southwest Medical University, Luzhou, 646000 China; 14 Clinical Research Institute, The Affiliated Hospital of Southwest Medical University, Luzhou, China; 15 School of Clinical Medical, Southwest Medical University, Luzhou, China; 16 Clinical Molecular Medicine Testing Center, Key Laboratory of Clinical Laboratory Diagnostics (Chinese Ministry of Education), The First Affiliated Hospital of Chongqing Medical University, Chongqing, 400016 China; 17 Department of Quantitative Health Sciences, John A. Burns School of Medicine, University of Hawaii at Manoa, Honolulu, Hawaii USA

**Keywords:** Cancer, Computational biology and bioinformatics, Gastroenterology, Oncology

## Abstract

This study aimed to integrate artificial intelligence (AI)with heat shock protein 90 alpha (HSP90α)expression to improve patient selection and prognostic assessment in unresectable hepatocellular carcinoma (HCC)treated with transarterial chemoembolization (TACE). We retrospectively enrolled 2555 unresectable HCC patients treated between 2016 and 2021 at seven Chinese tertiary hospitals. Residual-based methods were used to define TACE benefit. Eight AI models revealed that HSP90α expression, Barcelona Clinic Liver Cancer (BCLC)stage, and tumor size were key predictive factors for TACE benefit. A nomogram based on these three variables achieved an area under the receiver operating characteristic curve (AUC)of 0.901 in the validation cohort. For overall survival (OS), we developed 101 machine learning models. The StepCox[forward] plus random survival forest model showed the best performance. Its C-indices were 0.84, 0.70, and 0.78 in the training, internal validation, and external validation sets, respectively. In the internal validation set, the time-dependent AUCs for 1-, 2-, and 3 year OS were 0.835, 0.821, and 0.776; in the external validation set, they were 0.854, 0.790, and 0.804. Integrating AI with HSP90α enables robust identification of TACE-benefit candidates and accurate prognostic stratification in unresectable HCC.

## Introduction

Hepatocellular carcinoma (HCC)is one of the most common and aggressive malignancies worldwide, with a high incidence and mortality rate^1^. In most cases, HCC is diagnosed at advanced stages when surgical resection is no longer feasible due to tumor size, location, or liver function impairment^2^. Transarterial chemoembolization (TACE)has now become the most widely used local treatment for patients with unresectable HCC^3^. It is effective in controlling tumor growth and improving survival in many patients, making it a cornerstone in the management of intermediate-stage HCC^4^.

However, despite its widespread use, TACE is not suitable for all patients. Treatment options for liver cancer are diverse, including radiotherapy and systemic therapies, among others. The combination of local and systemic treatments has been shown to significantly improve survival outcomes compared to systemic treatment alone^5–7^. However, if a patient does not benefit from TACE, they may miss the opportunity to receive other potentially effective treatments. The benefit of TACE is not uniformly observed across all patients, highlighting the need for precise patient selection^8^. Identifying which patients are likely to benefit from TACE remains a critical challenge, as inappropriate treatment not only fails to improve survival but may also delay or prevent the application of alternative therapies.

In recent years, artificial intelligence (AI), particularly machine learning, has emerged as a powerful tool in medical research, especially in the development of predictive models for clinical decision-making^9,10^. Machine learning algorithms excel at analyzing complex patterns within large datasets, allowing for more accurate predictions of patient outcomes^11^. However, despite advancements in liver cancer diagnosis, a significant challenge remains in identifying reliable biomarkers for alpha-fetoprotein (AFP)-negative tumors, which account for 30–40% of pathologically diagnosed HCC patients^12^.

Heat shock protein 90 alpha (HSP90α)has recently emerged as a promising circulating biomarker in oncology, including HCC. HSP90α is a molecular chaperone that stabilizes and activates a wide range of oncogenic client proteins and regulates multiple cancer-related pathways, such as cell growth and survival, DNA damage response, angiogenesis, epithelial–mesenchymal transition, and cell migration. Tumor cells can also secrete extracellular HSP90α, which further promotes invasion, metastasis, and neovascularization in the tumor microenvironment^13^.In HCC, several clinical studies have shown that plasma HSP90α levels are significantly higher in patients with liver cancer than in healthy individuals or those with benign liver diseases, and that HSP90α levels correlate positively with tumor burden and disease severity, including larger tumor size, multiple lesions, portal vein tumor thrombosis, extrahepatic metastasis, advanced BCLC stage, elevated AFP, and impaired liver function^14^.Moreover, dynamic changes in plasma HSP90α have been reported to reflect tumor load and treatment response, with levels decreasing after tumor resection or effective therapy, and higher baseline HSP90α being associated with worse prognosis^15^. Our previous multicenter study also demonstrated that elevated HSP90α is independently associated with poorer survival and can serve as both a prognostic and predictive biomarker in HCC, making it an attractive candidate for integration into AI-based prediction models^16^.

This study aims to leverage machine learning techniques to identify patients who are most likely to benefit from TACE and predict the survival outcomes of patients with unresectable HCC undergoing TACE. By integrating machine learning with biomarker data, we hope to provide a more personalized approach to TACE treatment, improving patient outcomes and minimizing unnecessary interventions.

## Results

### Patient characteristics

**Fig. 1:**
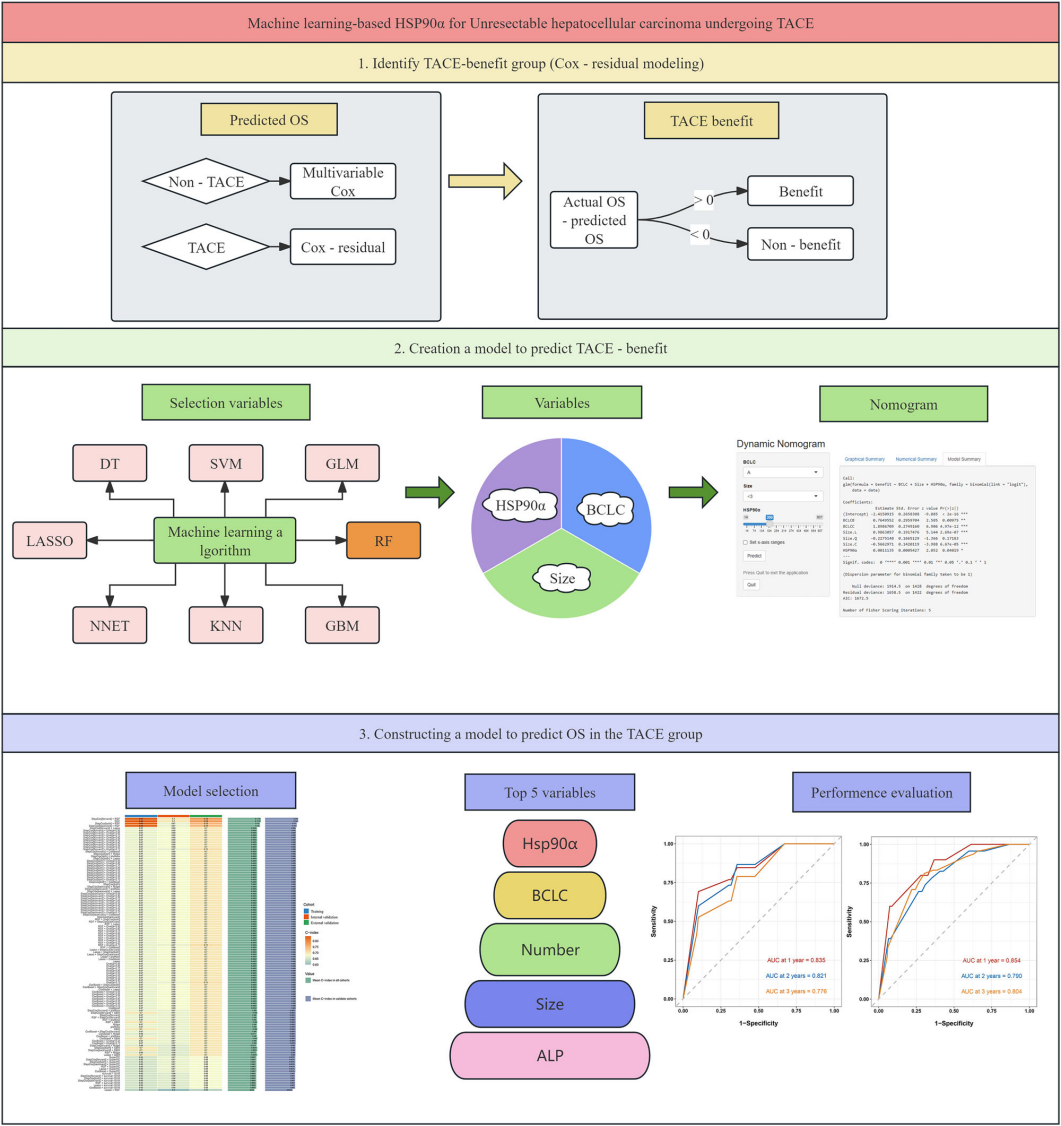
Workflow diagram of this study.

The technical workflow of this study is shown in Fig. 1. Before PSM, there were significant differences between the non-TACE (*n* = 1126)and TACE (*n* = 1429)groups in age, Hepatitis B Virus (HBV), diabetes mellitus, Child, ALBI (Albumin-bilirubin)grade, AFP, alkaline phosphatase (ALP), alanine aminotransferase (ALT), barcelona clinic liver Cancer (BCLC)stage, tumor number, tumor size, portal vein tumor thrombosis (PVTT), and metastasis (M, all *P* < 0.05). After 1:1 PSM, a total of 666 matched pairs of patients were identified. No significant differences in baseline characteristics were observed between the two groups (Table 1).

**Table. 1:** Baseline characteristics of the patients before and after PSM

		Before PSM			After PSM		
Variable	ALL	No TACE	TACE	P	No TACE	TACE	P
		N (%)	N (%)		N (%)	N (%)	
Patients	2555	1126	1429		666	666	
Male sex	2049 (80.2)	858 (76.2)	1191 (83.3)		546 (82.0)	544 (81.7)	
Age ≥ 65 years	666 (26.1)	339 (30.1)	327 (22.9)	<0.001	184 (27.6)	175 (26.3)	0.621
HSP90α (mean ± SD, ng/mL)	158 ± 118	158 ± 121	157 ± 116	0.930	156 ± 121	151 ± 114	0.385
HBV	1397 (54.7)	518 (46.0)	879 (61.5)	<0.001	360 (54.1)	374 (56.2)	0.474
Diabetes mellitus	240 (9.39)	126 (11.2)	114 (7.98)	0.007	66 (9.91)	68 (10.2)	0.927
Hypertension	360 (14.1)	163 (14.5)	197 (13.8)	0.660	101 (15.2)	104 (15.6)	0.879
Child				<0.001			0.721
A	1753 (68.6)	678 (60.2)	1075 (75.2)		466 (70.0)	459 (68.9)	
B	802 (31.4)	448 (39.8)	354 (24.8)		200 (30.0)	207 (31.1)	
ALBI grade				<0.001			0.728
1	656 (25.7)	281 (25.0)	375 (26.2)		169 (25.4)	180 (27.0)	
2	1627 (63.7)	643 (57.1)	984 (68.9)		444 (66.7)	438 (65.8)	
3	272 (10.6)	202 (17.9)	70 (4.90)		53 (7.96)	48 (7.21)	
AFP ≥ 400 ng/mL	1238 (48.5)	462 (41.0)	776 (54.3)	<0.001	311 (46.7)	299 (44.9)	0.545
ALP ≥ 125 U/L	1557 (60.9)	721 (64.0)	836 (58.5)	0.005	400 (60.1)	399 (59.9)	1.000
Platelet ≥ 100×109/L	1822 (71.3)	817 (72.6)	1005 (70.3)	0.233	475 (71.3)	472 (70.9)	0.904
ALT levels ≥ 40 U/L	1356 (53.1)	630 (56.0)	726 (50.8)	0.011	347 (52.1)	358 (53.8)	0.583
Leukocyte ≥ 4× 109/L	2099 (82.2)	944 (83.8)	1155 (80.8)	0.055	539 (80.9)	548 (82.3)	0.572
BCLC				<0.001			0.738
A	386 (15.1)	198 (17.6)	188 (13.2)		105 (15.8)	109 (16.4)	
B	485 (19.0)	154 (13.7)	331 (23.2)		114 (17.1)	123 (18.5)	
C	1684 (65.9)	774 (68.7)	910 (63.7)		447 (67.1)	434 (65.2)	
Tumor number ≥ 2	1977 (77.4)	838 (74.4)	1139 (79.7)	0.002	511 (76.7)	514 (77.2)	0.896
Tumor size, cm				<0.001			0.836
< 3	328 (12.8)	175 (15.5)	153 (10.7)		91 (13.7)	87 (13.1)	
≥3, <5	546 (21.4)	295 (26.2)	251 (17.6)		156 (23.4)	160 (24.0)	
≥5, <10	955 (37.4)	423 (37.6)	532 (37.2)		246 (36.9)	258 (38.7)	
≥10	726 (28.4)	233 (20.7)	493 (34.5)		173 (26.0)	161 (24.2)	
PVTT	985 (38.6)	391 (34.7)	594 (41.6)	<0.001	265 (39.8)	252 (37.8)	0.500
N	1248 (48.8)	568 (50.4)	680 (47.6)	0.163	332 (49.8)	320 (48.0)	0.547
M	642 (25.1)	349 (31.0)	293 (20.5)	<0.001	173 (26.0)	162 (24.3)	0.528

*PSM* propensity score matching, *HSP90α* heat-shock protein 90α, *HBV* hepatitis B virus, *ALBI* albumin–bilirubin, *AFP* alpha fetoprotein, *ALP* alkaline phosphatase, *ALT* alanine ami notransferase, *PVTT* portal vein tumor thrombus, *TACE* transcatheter arterial chemoembolization.

### Survival

**Fig. 2:**
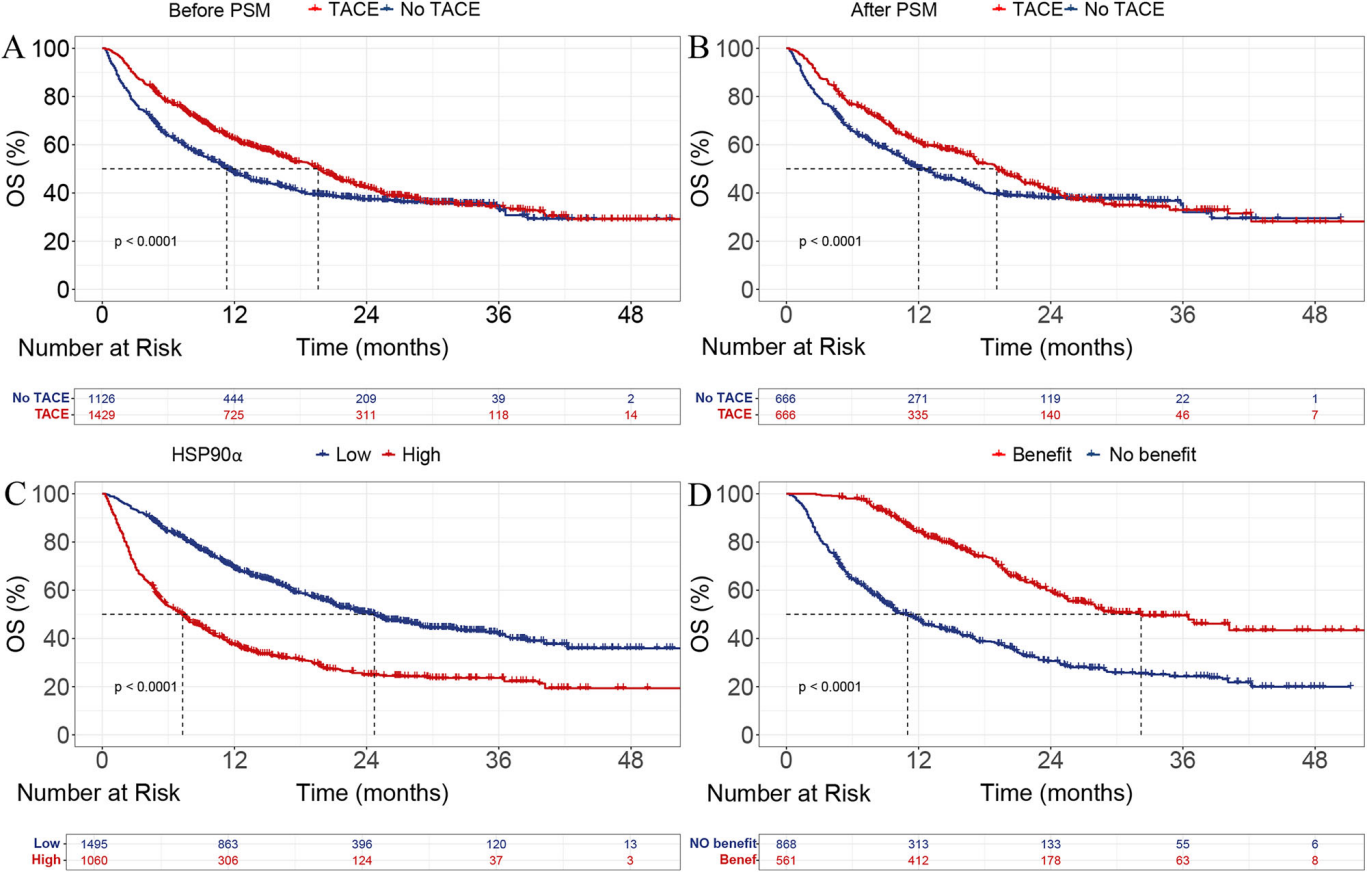
Kaplan–Meier curves for OS in different patient groups. **A** OS before PSM, comparing TACE and no TACE groups. **B** OS after PSM, comparing TACE and no TACE groups. **C** OS by HSP90α expression levels (low vs. high). **D** OS by treatment benefit (benefit vs. no benefit). OS, overall survival; PSM, propensity score matching; TACE, transarterial chemoembolization; HSP90α, heat shock protein 90 alpha.

Before propensity score matching (PSM), the median overall survival (mOS)in the TACE group was significantly longer than that in the non-TACE group (19.6 [18.4–21.0] vs. 11.3 [10.3–12.9] months, *P* < 0.001). The 1-, 2-, and 3 year OS rates in the TACE group were 62.3%, 42.2%, and 34.4%, respectively, whereas the corresponding rates in the non-TACE group were 47.8%, 37.3%, and 32.9% (Fig. 2A). After PSM, the TACE group still showed a superior mOS compared with the non-TACE group (19.1 [17.1–21.3] vs. 12.0 [10.8–14.4] months, *P* < 0.001; Fig. 2B).

Based on our previous research^16^, HSP90α levels ≥ 143.5 ng/mL were categorized into the high-expression group, while levels below this threshold were classified into the low-expression group. The mOS for the high-expression group was 7.3 (6.3-8.0)months, while the median OS for the low-expression group was 24.7 (22.5-27.7)months (*P* < 0.001, Fig. 2C).

### Machine learning prediction of TACE-benefit in the TACE group

Initially, the TACE benefit group was established using the residual method. The TACE benefit group exhibited a significantly longer mOS compared to the non-benefit group (32.2 (27.1-NA)vs. 11.0 (9.7-12.4)months, *P* < 0.001, Fig. 2D).

**Fig. 3:**
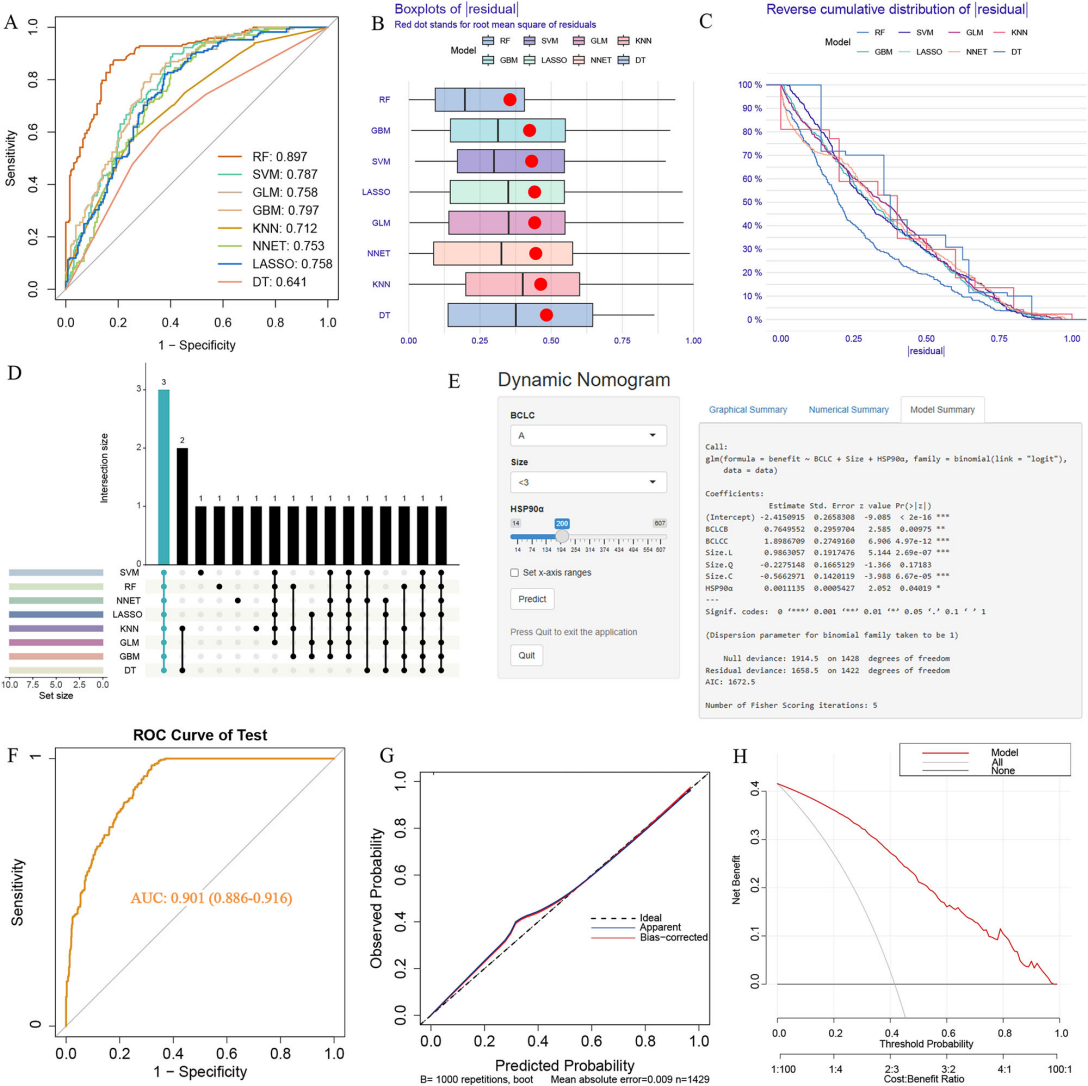
Development and validation of the TACE benefit prediction model. **A** ROC curve showing the RF model’s superior performance with an AUC-ROC of 0.897. **B** Residual boxplot indicating the smallest residuals in the RF model, reflecting better predictive performance. **C** Reverse cumulative distribution curve demonstrating the RF model’s superior prediction accuracy compared to other models. **D** Upset plot illustrating the intersection of the top 10 predictive factors from the 8 models, selecting three key factors: HSP90α, BCLC, and size. **E** Online Nomogram developed based on HSP90α, BCLC, and Size to predict TACE benefit in the training set. **F** ROC curve in the validation set, achieving an ROC value of 0.901 for predicting TACE benefit. **G** Calibration curve showing the model’s robust stability and prediction accuracy. **H** Decision Curve Analysis confirming the model’s clinical utility. AUC-ROC, area under the receiver operating characteristic curve; HSP90α, heat shock protein 90 alpha; BCLC, barcelona clinic liver Cancer; TACE, transarterial chemoembolization.

In the TACE group, LASSO (Least Absolute Shrinkage and Selection Operator), Random Forest (RF), Support Vector Machine (SVM), Generalized Linear Model (GLM), Gradient Boosting Machine (GBM), K-Nearest Neighbors (KNN), Neural Network (NNET), and Decision Tree (DT)were used to select factors associated with TACE benefit. The ROC curve shows that the RF model performs the best with an area under the receiver operating characteristic (AUC-ROC)of 0.897 (Fig. 3A).

The residual boxplot shows that the RF model has the smallest residuals, indicating better predictive performance (Fig. 3B). The reverse cumulative distribution curve further demonstrates that the RF model has superior prediction accuracy compared to other models (Fig. 3C). The variable importance plot reveals that HSP90α is the most influential factor in predicting TACE benefit within the RF model (Supplementary Fig. 1). The Upset plot shows the intersection of the top 10 predictive factors from the 8 models, ultimately selecting three factors: HSP90α, BCLC, and size (Fig. 3D).

A total of 1429 patients who underwent TACE were randomly divided into a training set (*n* = 999)and a validation set (*n* = 430)in a 7:3 ratio. Baseline characteristics are shown in Supplementary Table 1. There were no statistically significant differences in baseline characteristics between the training and validation sets. An online Nomogram (https://kesu.shinyapps.io/DynNomapp/)was developed to predict TACE benefit in the training set, based on three factors: HSP90α, BCLC, and Size (Fig. 3E). In the validation set, the model achieved an ROC value of 0.901 for predicting TACE benefit (Fig. 3F), and both the calibration curve (Fig. 3G)and decision curve analysis (DCA)curve (Fig. 3H)confirmed the model’s robust stability.

### Machine learning-based prediction of OS in the TACE group

In a cohort of 1429 unresectable HCC patients treated with TACE, no significant baseline differences were observed among the three groups: the training set (*n* = 790), the internal validation set (*n* = 340), and the external validation set (*n* = 299, Table 2).

**Table. 2:** Baseline Characteristics of Patients in the Training, Internal Validation, and External Validation Sets

Variable	Train	Internal validation	External validation	*P*
	*N* (%)	*N* (%)	*N* (%)	
Patients	790	340	299	
Male sex	659 (83.4)	286 (84.1)	246 (82.3)	0.82
Age ≥ 65 years	179 (22.7)	86 (25.3)	62 (20.7)	0.382
HSP90α (mean ± SD, ng/mL)	156 ± 116	161 ± 117	156 ± 116	0.781
HBV	480 (60.8)	209 (61.5)	190 (63.5)	0.701
Diabetes mellitus	61 (7.72)	27 (7.94)	26 (8.70)	0.869
Hypertension	121 (15.3)	43 (12.6)	33 (11.0)	0.147
Child				0.957
A	596 (75.4)	256 (75.3)	223 (74.6)	
B	194 (24.6)	84 (24.7)	76 (25.4)	
ALBI grade				0.733
1	216 (27.3)	89 (26.2)	70 (23.4)	
2	538 (68.1)	233 (68.5)	213 (71.2)	
3	36 (4.56)	18 (5.29)	16 (5.35)	
AFP ≥ 400 ng/mL	413 (52.3)	175 (51.5)	188 (62.9)	0.004
ALP ≥ 125 U/L	448 (56.7)	212 (62.4)	176 (58.9)	0.208
Platelet ≥ 100×10^9^/L	554 (70.1)	244 (71.8)	207 (69.2)	0.769
ALT levels ≥ 40 U/L	394 (49.9)	187 (55.0)	145 (48.5)	0.191
Leukocyte ≥ 4× 10^9^/L	644 (81.5)	275 (80.9)	236 (78.9)	0.625
BCLC				
A	98 (12.4)	46 (13.5)	44 (14.7)	0.651
B	177 (22.4)	79 (23.2)	75 (25.1)	
C	515 (65.2)	215 (63.2)	180 (60.2)	
Tumor number ≥ 2	629 (79.6)	279 (82.1)	231 (77.3)	0.321
Tumor size, cm				0.951
< 3	87 (11.0)	32 (9.41)	34 (11.4)	
≥3, <5	142 (18.0)	56 (16.5)	53 (17.7)	
≥5, <10	288 (36.5)	131 (38.5)	113 (37.8)	
≥10	273 (34.6)	121 (35.6)	99 (33.1)	
PVTT	328 (41.5)	141 (41.5)	125 (41.8)	0.995
N	392 (49.6)	163 (47.9)	125 (41.8)	0.069
M	157 (19.9)	63 (18.5)	73 (24.4)	0.149

*HSP90α* heat-shock protein 90α, *HBV* hepatitis B virus, *ALBI* albumin–bilirubin, *AFP* alpha fetoprotein; *ALP* alkaline phosphatase, *ALT* alanine ami notransferase, *PVTT* portal vein tumor thrombus.

**Fig. 4:**
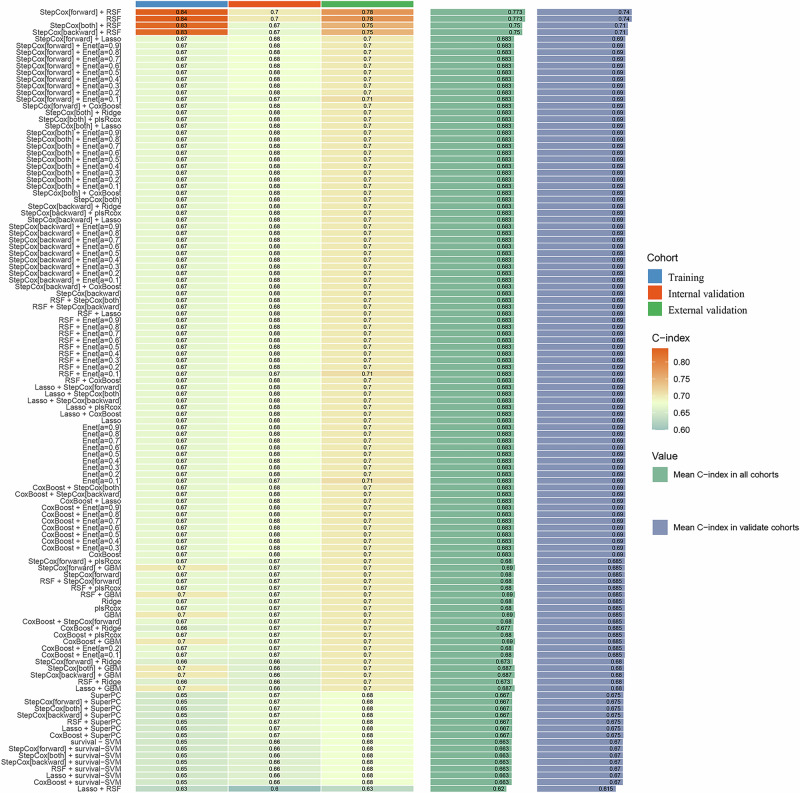
C-index of 101 machine learning models constructed in the training set, internal validation set, and external validation set.

First, univariate Cox regression analysis was conducted to identify 13 prognostic factors associated with OS, including HSP90α, Hypertension, Child, ALBI, AFP, ALP, ALT, BCLC, number of tumors, tumor size, PVTT, N, and M. Subsequently, 101 machine learning models were developed to predict OS in TACE group. Among these models, the StepCox[forward] + RSF model exhibited the highest C-index, with values of 0.84, 0.70, and 0.78 in the training set, internal validation set, and external validation set, respectively (Fig. 4). The StepCox[forward] + RSF model was developed by first using stepwise Cox regression to select significant prognostic factors, followed by applying the RSF model to predict overall survival.

Risk scores for TACE group were calculated based on the StepCox[forward] + RSF model. As shown in Supplementary Fig. 2, patients in the high-risk group had significantly shorter OS compared to those in the low-risk group (all *P* < 0.001), demonstrating the model’s strong predictive performance. The top five variables most strongly associated with OS were identified as HSP90α, BCLC, number of tumors, tumor size, and ALP.

**Fig. 5:**
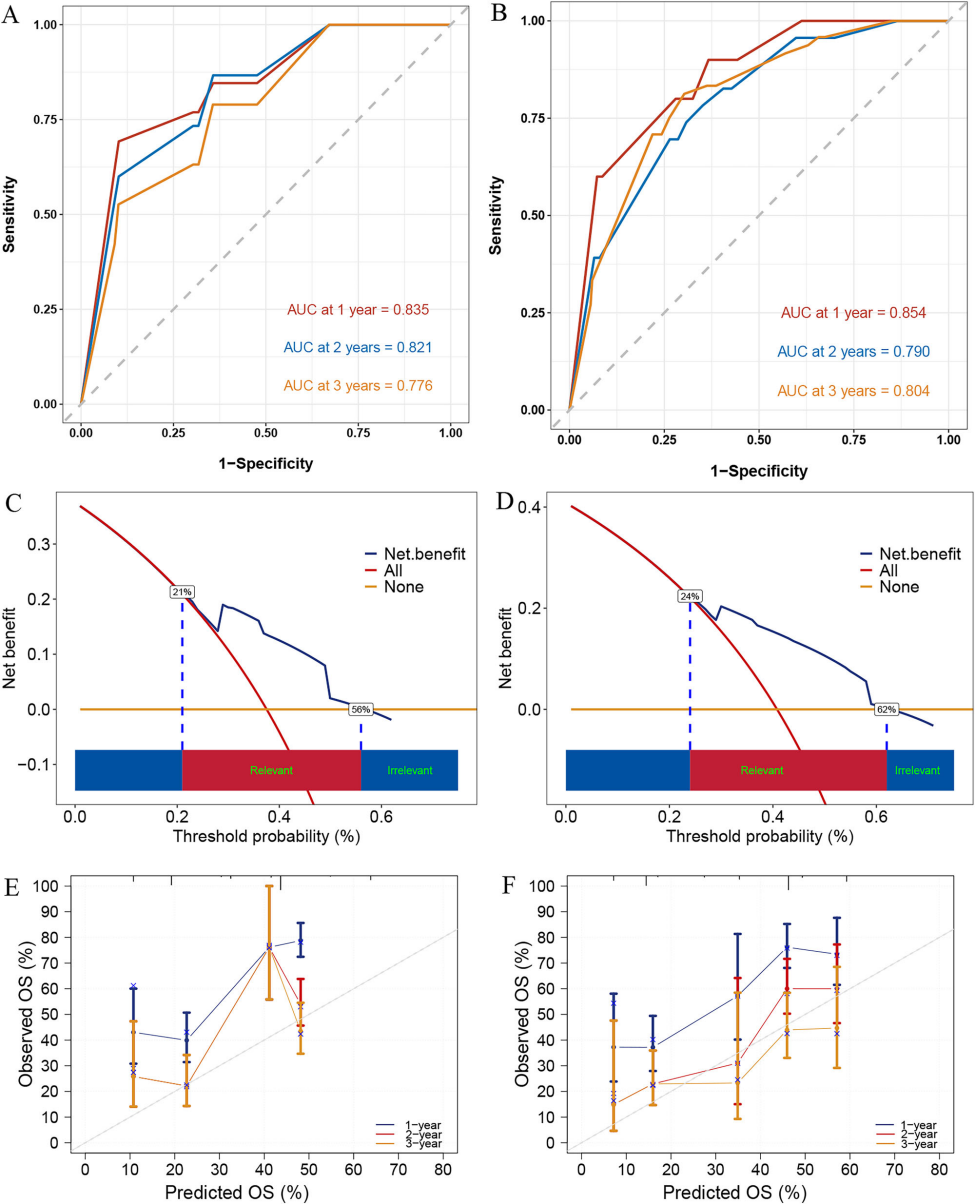
Performance evaluation of the StepCox[forward] + RSF model for predicting OS. The ROC curves for the internal (**A**) and external (**B**) validation sets, showing the AUC values for 1-, 2-, and 3 year OS. The decision curve analysis for the internal (**C**) and external (**D**) validation sets, with net benefits at varying threshold probabilities. Calibration plots for the internal (**E**) and external (**F**) validation sets, comparing predicted OS with observed OS. ROC, receiver operating characteristic curve; OS, overall survival.

The StepCox[forward] + RSF model was further validated in both the internal and external validation sets. The AUC-ROC values for predicting 1-, 2-, and 3 year OS in the internal validation set were 0.835, 0.821, and 0.776, respectively (Fig. 5A). In the external validation set, the AUC-ROC values were 0.854, 0.790, and 0.804 (Fig. 5B). The DCA demonstrated significant clinical benefit, with higher net benefits at low threshold probabilities (0-20%), outperforming both the “All treatment” and “No treatment” strategies (Fig. 5C, D). The calibration plot showed that the observed survival closely matched the predictions of the StepCox[forward] + RSF model, confirming its accuracy in predicting 1-, 2-, and 3 year OS. These results highlight the model’s consistency and reliability (Fig. 5E, F).

### HSP90α Expression and Clinical Factors

**Fig. 6:**
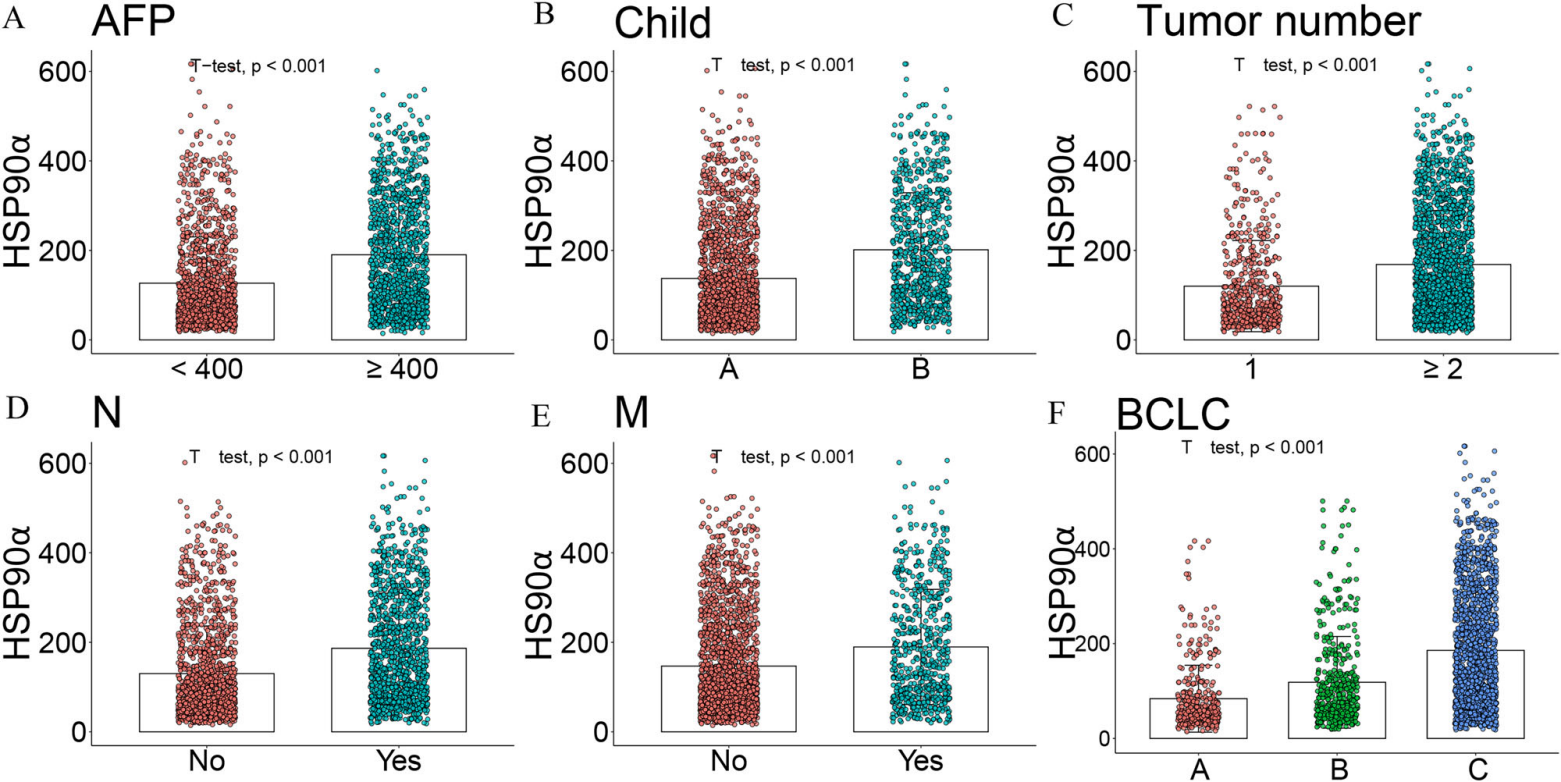
Association between HSP90α expression and key clinical characteristics. The relationship between HSP90α expression and AFP (**A**), Child-Pugh class (**B**), tumor number (**C**), N stage (**D**), M stage (**E**), and BCLC stage (**F**). HSP90α, heat shock protein 90 alpha; BCLC, barcelona clinic liver Cancer.

Based on baseline stratification, HSP90α expression was positively correlated with higher AFP (Fig. 6A), worse Child-Pugh scores (Fig. 6B), more tumor number (Fig. 6C), the presence of N (Fig. 6D) and M (Fig. 6E)stages, and poorer BCLC staging (Fig. 6F).

## Discussion

TACE is widely used in the treatment of advanced-stage HCC. This study is the first to combine machine learning and HSP90α to identify patients who will benefit from TACE and to assess OS, providing a more personalized approach to clinical decision-making.

Our results showed high ROC values, with the AUC-ROC values surpassing those in current studies on HCC^17,18^. This indicates that our machine learning-based model, incorporating HSP90α as a predictive factor, is highly effective in identifying TACE benefit. Compared to other models in the literature, our study demonstrates superior accuracy in predicting OS at multiple time points, which can be attributed to the inclusion of HSP90α, a promising biomarker in HCC^19,20^.

Given the relatively low positive rate of AFP in some HCC cases, especially in AFP-negative patients, exploring alternative biomarkers is essential^12,13^. This study confirms that HSP90α expression is associated with worse OS and more advanced disease stages. Elevated levels of HSP90α correlate with poorer prognosis in HCC, and this protein plays a significant role in promoting cancer progression and resistance to treatment. It is involved in stabilizing various client proteins that are critical for tumor survival and metastasis, making it a potential therapeutic target for future treatment strategies^21–23^.

In this study, we initially screened HSP90α, BCLC, and tumor size as predictive factors for TACE benefit using eight different machine learning models. After selecting the most relevant features, we constructed an online nomogram, which achieved an AUC-ROC value of 0.901, confirming the model’s strong predictive performance. By combining machine learning-based feature selection with the nomogram, we developed a tool that allows for personalized risk assessment, helping clinicians identify patients most likely to benefit from TACE. This approach not only enhances the accuracy of treatment decisions but also minimizes the risk of administering ineffective treatments, thereby avoiding unnecessary interventions and ensuring that patients receive the most appropriate and beneficial therapy for their condition^24–26^.

Building upon this, we developed 101 machine learning models to further assess OS in patients with unresectable HCC undergoing TACE. Among these models, the StepCox[forward] + RSF model demonstrated the best predictive performance, with a C-index exceeding 0.8. In both the internal and external validation cohorts, the ROC for predicting 1-, 2-, and 3 year OS exceeded 0.8. The superior predictive accuracy of the StepCox[forward] + RSF model can be attributed to its ability to handle complex relationships between variables and its robust calibration. The forward selection process in StepCox ensures that only the most significant predictors are retained, while the RSF model contributes by incorporating the non-linear interactions between factors, making it particularly effective in heterogeneous data sets like those in HCC^27,28^.

Moreover, HSP90α was confirmed as one of the most important factors influencing OS in our model. Its inclusion not only improved the accuracy of survival prediction, but also highlighted the critical role of HSP90α in HCC prognosis. Elevated HSP90α expression has been consistently linked to worse survival and more advanced disease stages, supporting its potential as both a predictive biomarker and a therapeutic target. In our cohort, higher HSP90α levels were associated with larger tumors, more advanced stage, PVTT, metastasis, and impaired liver function. This pattern is biologically plausible, as elevated HSP90α likely reflects greater tumor burden, more aggressive tumor biology, and systemic inflammation in advanced HCC, and thus serves as an integrated marker of overall disease severity^29^. These findings reinforce the value of incorporating molecular markers such as HSP90α into predictive models to refine individualized treatment planning and risk stratification.

Numerous studies have shown that combining TACE with systemic treatments can provide superior survival benefits compared with either modality alone^30–32^. Because TACE remains the most commonly used locoregional treatment for unresectable HCC, accurately identifying which patients are likely to benefit from it is crucial. By integrating our TACE-benefit nomogram with the StepCox[forward] + RSF survival model, we can both identify patients who are more likely to derive benefit from TACE and quantify their expected OS under a TACE-based strategy. This combined approach has substantial potential to enhance the clinical management of unresectable HCC. By avoiding TACE in patients with a low predicted benefit, clinicians may reduce unnecessary procedures and facilitate timely transition to alternative systemic or trial-based therapies, thereby improving overall treatment efficiency and outcomes.

From a clinical perspective, the TACE-benefit nomogram and the StepCox[forward] + RSF survival model are designed to complement, rather than replace, multidisciplinary decision-making. The nomogram, which is available as an online calculator, can be applied at the time of initial treatment planning to estimate an individual patient’s probability of benefit from TACE based on HSP90α, BCLC stage, and tumor size. In practice, patients with a high predicted probability of TACE benefit could be prioritized for TACE-based strategies, whereas those with a low predicted probability might be considered for alternative locoregional approaches, upfront systemic therapy, or early enrolment in clinical trials. Patients with intermediate probabilities would be suitable for shared decision-making, taking into account liver function, comorbidities, patient preferences, and institutional expertise.

The StepCox[forward] + RSF survival model further refines risk stratification among TACE-treated patients by providing an individualized survival prediction and a model-based risk score. Using the predefined cut-offs employed in this study, patients in the low-risk group achieved substantially longer OS than those in the high-risk group. In real-world practice, low-risk patients with a high predicted probability of TACE benefit might be managed with TACE-based regimens combined with targeted and/or immunotherapy and standard surveillance intervals. In contrast, high-risk patients, particularly those with a low predicted TACE benefit, could be considered for earlier transition to systemic therapy, more intensive follow-up, or enrolment in trials testing novel combinations rather than repeated TACE. For example, a patient with intermediate-stage disease, limited tumor burden, preserved liver function, and low HSP90α levels may have both a high predicted probability of TACE benefit and a low-risk survival score, supporting TACE combined with targeted therapy as the initial strategy. Conversely, a patient with multifocal advanced-stage HCC, high HSP90α levels, and a high-risk score may have a low predicted TACE benefit, and in such a case the model outputs would favor upfront systemic therapy or clinical trial enrolment instead of repeated TACE.

However, this study has several limitations. First, although it was conducted across multiple centers, variations in clinical practice patterns and technical expertise between institutions may have affected the consistency of the findings. Second, although all patients received TACE-based therapy combined with targeted agents and, in a subset of cases, immunotherapy, the specific systemic regimens (including drug type, combination strategy, and dosing schedules)were not fully standardized across centers. We did not stratify our analyses according to individual targeted or immunotherapy regimens, and residual confounding from this treatment heterogeneity may have influenced effect estimates and model performance; however, this variation also reflects real-world clinical practice and may, to some extent, enhance the generalizability of our models. Third, the retrospective design of the study could introduce selection bias, despite the use of propensity score matching to control for confounding variables. Future prospective, multicenter studies are needed to validate our findings and further refine the predictive model. Moreover, the mechanism by which HSP90α influences TACE outcomes requires further investigation through laboratory studies to better understand its role in tumor biology and treatment resistance. Finally, all patients in this study were treated at tertiary centers in China, where the etiology of HCC is predominantly HBV-related and local practice patterns for TACE and systemic therapy may differ from those in Western countries or regions. As a result, the performance and clinical utility of our models may not be fully generalizable to populations with different etiologic profiles, liver function reserves, or treatment availability. External validation in independent cohorts from other geographic regions and healthcare systems, and, if necessary, model recalibration or retraining, will be essential before these tools can be widely adopted in non-Chinese settings^33–35^.

This study demonstrates that integrating AI with HSP90α expression can identify unresectable HCC patients who are more likely to benefit from TACE and provide robust prediction of OS. The TACE-benefit nomogram and StepCox[forward] + RSF survival model offer a practical framework to support personalized treatment selection and to avoid unnecessary or ineffective TACE in real-world practice.

## Methods

### Patients

A total of 2555 unresectable HCC patients were initially enrolled at seven Chinese tertiary hospitals between 2016 and 2021. Inclusion criteria were: (1) clinically or pathologically diagnosed HCC, (2) Child-Pugh class A or B, (3) at least one measurable lesion according to the Response Evaluation Criteria in Solid Tumors (RECIST), and (4) available pre-treatment HSP90α data. Exclusion criteria included: (1) presence of other malignant tumors, (2) contraindications to TACE, such as severe cirrhotic ascites, active infections, or poor liver function, (3) incomplete clinical information, and (4) loss to follow-up.

Serum HSP90α levels were measured independently at the clinical laboratories of each participating hospital according to routine institutional procedures.

HCC was clinically diagnosed by experienced clinicians when the following criteria were met^1^: presence of liver cirrhosis with chronic hepatitis B and/or hepatitis C virus infection and a newly detected hepatic mass on imaging^2^; a hepatic lesion ≥ 2 cm in diameter showing typical HCC enhancement on contrast-enhanced CT or MRI (heterogeneous arterial phase hyperenhancement with rapid washout in the portal venous or delayed phase)^3^; for hepatic lesions < 2 cm, the same typical enhancement pattern observed on both contrast-enhanced CT and MRI; or^4^ persistently elevated serum AFP, defined as ≥ 400 μg/L for ≥ 1 month or ≥ 200 μg/L for ≥ 2 months, in the presence of a compatible hepatic mass on imaging.

This study was approved by the Ethics Committee of Chongqing People’s Hospital (KY S2025-029-01)and was conducted in accordance with the principles outlined in the Declaration of Helsinki. All patients provided written informed consent prior to receiving treatment, and the study adhered to ethical guidelines for medical research.

### Treatment and follow-up

TACE was carried out with the assistance of a digital subtraction angiography (DSA)system. The procedure commenced with the Seldinger technique to access the celiac trunk and superior mesenteric artery, allowing for precise catheter-based angiographic imaging to assess the tumor’s vascular supply and extent. Following this, a microcatheter was navigated into the artery providing blood to the tumor, where a combination of chemotherapy agents and embolic materials were delivered. Once successful embolization was verified by angiography, the catheter and sheath were removed, and hemostatic pressure was applied to the puncture site using bandages. All patients received targeted therapy, and a subset of patients also received immunotherapy as part of their treatment regimen.

The primary endpoint was OS, which was measured from the start of treatment until death from any cause or the last follow-up for censored individuals.

### Cox-residual modeling

To identify patients who were most likely to benefit from TACE, we used a Cox–residual strategy. First, a multivariable Cox proportional hazards model for OS was fitted in the non-TACE cohort. For each patient in the TACE cohort, this Cox model was then used to calculate an individual linear predictor based on their baseline characteristics and to derive a model-based expected OS. The Cox residual for each TACE-treated patient was defined as the difference between the observed OS and the expected OS (residual = observed OS − expected OS). Patients with positive residuals, indicating that their observed survival exceeded the model-predicted survival, were classified as the “TACE benefit” group, whereas those with zero or negative residuals were classified as the “non-benefit” group.

### Machine learning models for TACE benefit

A total of 1429 patients who underwent TACE were randomly divided into a training set (*n* = 999)and a validation set (*n* = 430)in a 7:3 ratio. Eight machine learning algorithms, including LASSO, RF, SVM, GLM, GBM, KNN, NNET, and DT models, were applied to identify predictive indicators of TACE benefit. For all machine-learning models, hyperparameters were selected using 10-fold cross-validation.

An UpSet plot was then used to extract the intersecting indicators across the eight models, thereby screening out the most robust predictors. Based on these intersection indicators, an online nomogram was developed in the training set. The performance of the nomogram was subsequently evaluated in the validation set using ROC curves, calibration curves, and DCA.

### Machine learning models for OS in TACE group

1130 HCC patients treated with TACE from 6 hospitals were randomly split into a training set (*n* = 790)and an internal validation set(*n* = 340)in a 7:3 ratio. In addition, data from 299 unresectable HCC patients who underwent TACE at the Affiliated Hospital of Southwest Medical University were used as an independent external validation set.

In the training set, univariate Cox regression analysis was first performed to identify prognostic indicators in unresectable HCC patients receiving TACE. Subsequently, a total of 101 machine learning models were constructed to predict OS, and the model with the highest concordance index (C-index)was selected. In both the internal and external validation sets, ROC curves, calibration curves, and DCA were employed to evaluate the predictive performance of the selected model for 1-, 2-, and 3 year OS.

### Statistical analysis

Categorical variables were processed using chi-square tests or Fisher’s exact tests, while continuous variables were handled using *t*-tests for normally distributed data or Mann-Whitney *U* tests for non-normally distributed data. PSM was used to eliminate baseline differences between the non-TACE and TACE groups. Propensity scores were estimated using a logistic regression model based on baseline covariates, and patients in the two groups were then matched in a 1:1 ratio using nearest-neighbor matching without replacement with a caliper of 0.1 on the propensity score. Kaplan-Meier (KM)curves were used to assess OS, and differences between groups were calculated using the log-rank test. All statistical analyses were conducted using R software. A *p*-value of <0.05 was considered statistically significant.

## Supplementary information

Supplementary Information

## Data Availability

All data generated or analyzed during this study are included in this article. Further enquiries can be directed to the corresponding author (cqghxuke@cqu.edu.cn).

## Code availability

All code is available from the corresponding author upon reasonable request (cqghxuke@cqu.edu.cn).
