# Supplementary material for: Artificial intelligence-based personalized treatment strategies for unresectable hepatocellular carcinoma: integrating HSP90α for prognosis and survival prediction
Source: NPJ Digit Med. 2025 Dec 27;9:94. doi: 10.1038/s41746-025-02281-y (PMC12855880; doi:10.1038/s41746-025-02281-y)

Supplementary Table 1. Baseline Characteristics of Patients in the Training and Validation Sets

Variable	TACE<br>N (%)	Train<br>N (%)	Validation<br>N (%)	P
Patients	1429	999	430	
Male sex	1191 (83.3)	828 (82.9)	363 (84.4)	0.524
Age ≥ 65 years	327 (22.9)	233 (23.3)	94 (21.9)	0.593
HSP90α (mean ± SD, ng/mL)	157 ± 116)	159 ± 116	154 ± 117	0.471
HBV	879 (61.5)	602 (60.3)	277 (64.4)	0.155
Diabetes mellitus	114 (7.98)	73 (7.31)	41 (9.53)	0.187
Hypertension	197 (13.8)	126 (12.6)	71 (16.5)	0.060
Child				0.891
A	1075 (75.2)	750 (75.1)	325 (75.6)	
B	354 (24.8)	249 (24.9)	105 (24.4)	
ALBI grade				0.375
1	375 (26.2)	252 (25.2)	123 (28.6)	
2	984 (68.9)	699 (70.0)	285 (66.3)	
3	70 (4.90)	48 (4.80)	22 (5.12)	
AFP ≥ 400 ng/mL	776 (54.3)	559 (56.0)	217 (50.5)	0.064
ALP ≥ 125 U/L	836 (58.5)	586 (58.7)	250 (58.1)	0.901
Platelet ≥ 100×10^9/L	1005 (70.3)	690 (69.1)	315 (73.3)	0.127
ALT levels ≥ 40 U/L	726 (50.8)	516 (51.7)	210 (48.8)	0.358
Leukocyte ≥ 4×10^9/L	1155 (80.8)	802 (80.3)	353 (82.1)	0.468
BCLC				0.149
A	188 (13.2)	128 (12.8)	60 (14.0)	
B	331 (23.2)	219 (21.9)	112 (26.0)	
C	910 (63.7)	652 (65.3)	258 (60.0)	
Tumor number ≥ 2	1139 (79.7)	799 (80.0)	340 (79.1)	0.748
Tumor size, cm				0.375
< 3	153 (10.7)	111 (11.1)	42 (9.77)	
≥3, <5	251 (17.6)	165 (16.5)	86 (20.0)	
≥5, <10	532 (37.2)	371 (37.1)	161 (37.4)	
≥10	493 (34.5)	352 (35.2)	141 (32.8)	
PVTT	594 (41.6)	426 (42.6)	168 (39.1)	0.231
N	680 (47.6)	494 (49.4)	186 (43.3)	0.056
M	293 (20.5)	211 (21.1)	82 (19.1)	0.418

HSP90α, heat-shock protein 90α; HBV, hepatitis B virus; ALBI, albumin–bilirubin; AFP, alpha fetoprotein; ALP, alkaline phosphatase; ALT, alanine ami notransferase; PVTT, portal vein tumor thrombus; TACE, transcatheter arterial chemoembolization,

Supplementary Fig. 1. Variable importance plot based on the RF model for predicting TACE benefit.

TACE, transarterial chemoembolization.

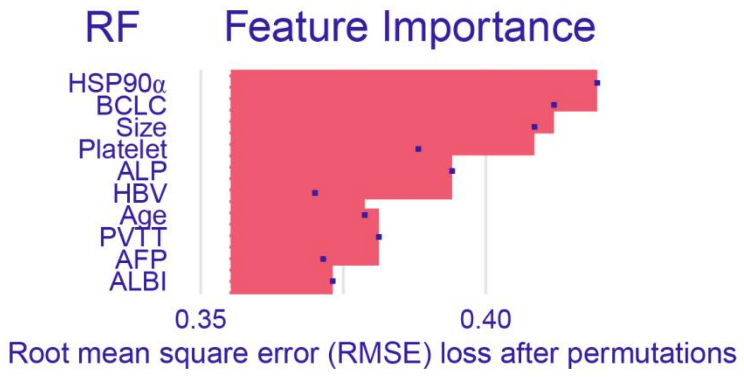

Supplementary Fig. 2. Patients in the high-risk group had significantly shorter OS compared to those in the low-risk group in the training set, internal validation set, and external validation set.

OS, overall survival.

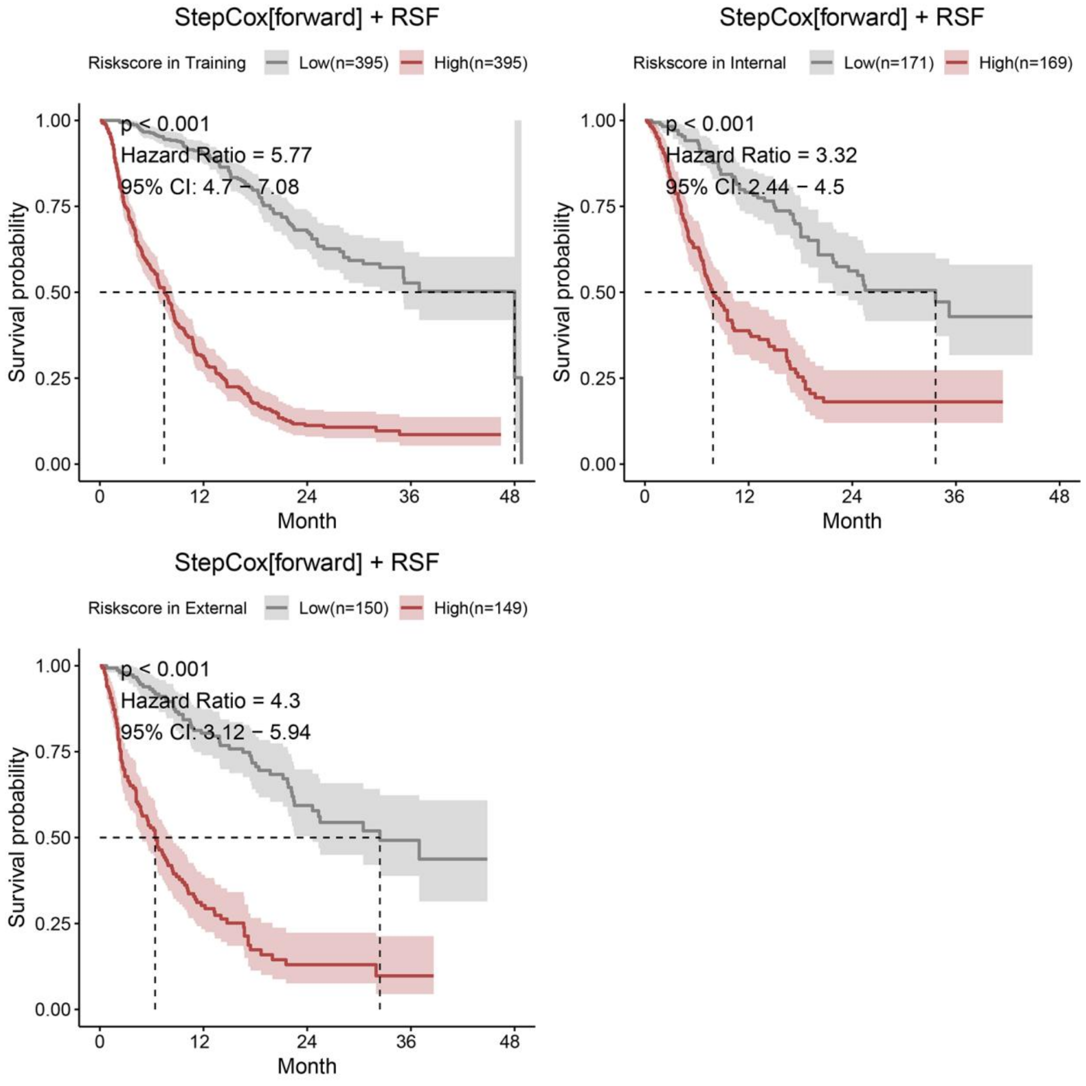

Supplement: Supplementary file 1 — Supplementary Information [file 41746_2025_2281_MOESM1_ESM.pdf]
